# The Impact of EGDT on Sepsis Mortality in a Single Tertiary Care Center in Lebanon

**DOI:** 10.1155/2019/8747282

**Published:** 2019-01-15

**Authors:** Christopher El Khuri, Gilbert Abou Dagher, Ali Chami, Ralph Bou Chebl, Tarek Amoun, Rana Bachir, Batoul Jaafar, Nesrine Rizk

**Affiliations:** ^1^Department of Emergency Medicine, American University of Beirut Medical Center, Beirut, Lebanon; ^2^Department of Internal Medicine, American University of Beirut Medical Center, Beirut, Lebanon; ^3^Division of Infectious Diseases, American University of Beirut Medical Center, Beirut, Lebanon

## Abstract

**Background:**

EGDT (Early Goal Directed Therapy) or some portion of EGDT has been shown to decrease mortality secondary to sepsis and septic shock.

**Objective:**

Our study aims to assess the effect of adopting this approach in the emergency department on in-hospital mortality secondary to sepsis/septic shock in Lebanon.

**Hypothesis:**

Implementation of the EGDT protocol of sepsis in ED will decrease in-hospital mortality.

**Methods:**

Our retrospective study included 290 adult patients presenting to the ED of a tertiary center in Lebanon with severe sepsis and/or septic shock. 145 patients between years 2013 and 2014 who received protocol care were compared to 145 patients treated by standard care between 2010 and 2012. Data from the EHR were retrieved about patients' demographics, medical comorbidities, and periresuscitation parameters. A multivariate analysis using logistic regression for the outcome in-hospital mortality after adjusting for protocol use and other confounders was done and AOR was obtained for the protocol use. 28-day mortality, ED, and hospital length of stay were compared between the two groups.

**Results:**

The most common infection site in the protocol arm was the lower respiratory tract (42.1%), and controls suffered more from UTIs (33.8%). Patients on protocol care had lower in-hospital mortality than that receiving usual care, 31.7% versus 47.6% (p=0.006) with an AOR of 0.429 (p =0.018). Protocol patients received more fluids at 6 and 24 hours (3.8 ± 1.7 L and 6.1 ± 2.1 L) compared to the control group (2.7 ± 2.0 L and 4.9 ± 2.8 L p=<0.001). Time to and duration of vasopressor use, choice of appropriate antibiotics, and length of ED stay were not significantly different between the two groups.

**Conclusion:**

EGDT- (Early Goal Directed Therapy-) based sepsis protocol implementation in EDs decreases in-hospital mortality in developing countries. Adopting this approach in facilities with limited resources, ICU capabilities, and prehospital systems may have a pronounced benefit.

## 1. Introduction

Sepsis is a life-threatening organ dysfunction caused by a dysregulation of host responses to infection [1]. Despite significant medical progress, it remains an often fatal condition with mortality reaching 25% [2]. Sepsis accounts for a large number of Emergency Department (ED) visits in the United States [3], and leads to more than half of in-hospital mortality [4]. A delay in initiating antibiotic therapy and fluid therapy is detrimental in sepsis management [5, 6].

In 2001, the early goal directed trial EGDT [7] introduced a 6-hour-protocol approach that resulted in a dramatic reduction in mortality from sepsis. This lead to the establishment of the Surviving Sepsis Campaign regularly updating sepsis management guidelines [1]. Recently three trials, ARISE, PROMISE, and Process, challenged the EGDT and showed similar survival with standard care [8–10], and this result was replicated by PRISM, a meta-analysis published in 2016 [11]. These trials were conducted in developed countries [11, 12] with rare studies exploring sepsis management in developing countries where hospital and human resources are limited [13]. Studies from Africa and the Middle East suggest a decreased mortality with the use of EGDT [14].

The primary outcome of our study is the 72 hours, hospital, and 28-day mortality to evaluate the implications of a structured sepsis protocol derived from the 2001 EGDT in patients presenting to the emergency department of a single tertiary hospital in Lebanon.

Secondary outcomes includes length of stay, time to antibiotics, percent of ICU admission, amount of IV fluid use, and vasopressor use.

## 2. Methods

### 2.1. Study Design and Patient Selection

This is an IRB approved, single center, retrospective, cohort study conducted in a large tertiary care center in Lebanon. We reviewed the electronic health record using the ICD-9 coding system of patients presenting to the emergency department with severe sepsis or septic shock. Patients who were eligible for the study should meet the definition of severe sepsis or septic shock as per the 2012 Surviving Sepsis Campaign guidelines definitions [15]. Severe sepsis was defined as sepsis with associated organ dysfunction with one of the following: SBP <90mmHg or MAP <65mmHg or lactate >2mmol/L after an initial fluid challenge; INR >1.5 or a PTT >60s; bilirubin >2 *μ*mol/L; urine output <0.5ml/kg/hr for 2hrs; creatinine>2mg/dl; platelet count <100,000/mm^3^; SpO_2_ <90%. Septic shock was defined as sepsis with any of the following: SBP <90mmHg or MAP <65mmHg despite 30ml/kg of crystalloid resuscitation (i.e., vasopressor dependence). We excluded patients younger than 18 years of age, pregnant, or presenting with cardiac arrest or trauma.

We included 145 consecutive patients from January 2013 until May 2014 presenting to our ED with severe sepsis and/or septic shock who were managed by the EGDT sepsis based protocol, and these constituted the intervention arm. For the control cohort, 145 patients with sepsis and/or septic shock presenting to the ED between January 2010 and December 2012 were randomly selected from a large pool of database using computer software for random number generation. The AUBMC-ED volume of patients diagnosed with severe sepsis and started on the protocol during the years following implementation of a sepsis protocol (2012-2014) was used to determine the size of the study population. A rough estimate of each group size ranges between 200 and 300 patients.

Medical records were used to retrieve data about patients' demographics including patients' age, gender and medical information regarding presence/absence of comorbidities, site of infection, microbiology findings, and site of disposition (intensive care unit ICU, general practice unit GPU or home). Pertinent laboratory blood workup and vital signs upon presentation and the latter 6 hours after resuscitation were also collected. Antibiotics use and its appropriateness (defined as initial antibiotic regimen used in the first 48hrs of treatment that matches the bacteria later recovered) were extracted. Data on the requirements of intravenous fluid resuscitation, vasopressor (use and duration) and steroid use were also retrieved. We also collected the length of stay in the ED, GPU and ICU along with in-hospital and 72-hr mortality.

### 2.2. EGDT-Sepsis Based Protocol Definition

For the intervention cohort, patients were managed as per our ED sepsis protocol (Supplementary Materials available here), that is, based on the EGDT recommendations [7].

### 2.3. Usual Care Definition

For the control cohort, the management was not standardized and was left as per the treating the physician, guided by individual preference practice.

### 2.4. Statistical Analysis

Statistical analyses were performed using SPSS version 24.0 (Armonk, NY: IBM Corp).

Univariate analysis was carried out between the intervention and the control groups for comparison of patients' characteristics, preresuscitation parameters, resuscitation parameters, and length of hospital stay/mortality outcomes. Comparison was done between patients' demographics, comorbidities, severity of sepsis, site of infection, and microbiology isolate (Table 1). Preresuscitation parameters included vital signs and pertinent laboratory findings upon presentation (Table 2). As for the parameters of resuscitation, they included patients' vital signs 6 hours after ED management, requirements of intravenous fluids at 6 and 24 hours, vasopressor/inotrope (use, time to start, and duration of use within the first 24 hours), CVC placement, and requirement of mechanical ventilation (Table 3). As for length of hospital stay (in ED, ICU, and GPU and in-hospital), 72hours and 28 days mortality were compared between the two arms (Table 4). For continuous variables, an independent t-test comparing the mean across both groups was done and both, the mean and standard deviation, are shown. For categorical variables, a chi-square test was run and data is represented as frequency percentages.

A multivariate analysis was performed using logistic regression to find the best model that fits the data and explains the use of the EGDT-sepsis based protocol as predictor of in-hospital mortality while controlling for all possible confounders. A backward selection procedure, with significance level for removal from the model set at 0.1, was conducted by fitting in-hospital mortality with all risk factors found to be significant in the bivariate level, in addition to those considered as being clinically meaningful. The included variables were protocol use, age, gender, diagnosis (severe sepsis or septic shock), systolic congestive heart failure (CHF) EF<40%, diabetes mellitus (DM), coronary artery disease (CAD), hypertension (HTN), cerebrovascular accidents (CVA), chronic kidney disease (CKD), hemodialysis (HD), chronic obstructive pulmonary disease (COPD), central venous catheter (CVC) placement, endotracheal tube placement, MAP upon presentation to the ED, BUN, creatinine, and appropriate use of antibiotics.

## 3. Results

### 3.1. Patients Characteristics

Two hundred and ninety patients were included in the final analysis with 145 patients in each arm. The protocol arm had a mean age of 71.9 ± 14.1 years compared to 72.9 ± 16.3 years in the control arm. 51.7% and 52.4% of the protocol group and control group, respectively, were male patients. All baseline demographics such as age, gender, and studied comorbidities in addition to diagnosis of severe sepsis or septic shock are presented (Table 1). The most common sites of infection in the protocol arm were respiratory (42.1%), urinary (35.9%) and gastrointestinal (14.5%) tracts compared to the control arm that had urinary (33.8%), respiratory (31.0%) tracts, and skin (13.1%). Both cohorts did not differ significantly in regards to the microbiology results except for enterococcus being more prevalent in the control arm (Table 1).

### 3.2. Pre-Resuscitation Parameters

There was no statistically significant difference between the two groups in terms of temperature, O_2_ saturation, SBP, or respiratory rate. Protocol patients had higher diastolic blood pressure (DBP=59.2 ± 16.3 mmHg vs. 54.7 ±16.0 mmHg p=0.019) and mean arterial pressure (MAP= 73.7 ±17.3 mmHg vs. 69.5 ± 17.6 mmHg p=0.043) and heart rate (HR= 106.2 ±24.2 bpm vs. 99.4 ± 25.3 bpm). When comparing initial laboratory results, most results were comparable between the groups except for creatinine (2.0 ± 1.4 mg/dl vs. 2.8 ± 2.2 mg/dl in the protocol and control groups, respectively, p=<0.001) and BUN (41.9 ± 29.9 mg/dl vs. 55.9 ± 39.5 mg/dl in the protocol and control groups, respectively, p=0.001). Lactate levels did not differ between the two groups (3.9 ± 2.5 mg/dl in the intervention cohort versus 3.8 ± 4.1 in the control cohort). Table 2 includes those parameters upon presentation to the ED.

### 3.3. Resuscitation Parameters

All vital signs were comparable between the groups after treatment, except for MAP where protocol patients had a lower MAP than the control group at 6 hours (69.8 ± 13.8 mmHg vs. 73.9 ± 14.9 mmHg, respectively, p=0.022). Patients who were managed using the sepsis protocol received more fluids at 6 and 24 hours (3.8 ± 1.7 L and 6.1 ± 2.1 L) compared to the control group (2.7 ± 2.0 L and 4.9 ± 2.8 L) p=<0.001. More vasopressors were initiated within the first 24 hours in the protocol arm as well (88 (60.6%) vs. 52 (35.8%) p=<0.001). The intervention group had more CVC placed (21.2% vs. 9.9% p=0.010). Both protocol and control cohorts have similar rates of mechanical ventilation. The time to vasopressor initiation and the duration of vasopressor use were not significantly different between the cohorts. Both cohorts had similar steroid and antibiotic use. The mean time of antibiotics initiation was 2.0 ± 3.6 hours in the protocol group compared to 2.8 ± 2.6 hours in the control group p=0.054. More antibiotics were initiated in the ED in the control arm than the protocol arm (99.3% vs. 93.8% p=0.010). Ninety-one patients (97.8%) in the intervention group received appropriate antibiotics compared to 75 (91.5%) in the control group (p=0.056). Table 3 summarizes the vital signs 6 hours after treatment and the general parameters of the resuscitation.

### 3.4. Length of Stay and Mortality Analysis

The length of stay in the ED was found to be 26.0 ± 29.0 hours vs. 19.4 ± 28.8 hours in the protocol and control group, respectively (p=0.051). ICU and GPU LOS did not differ between the two arms (Table 4). Forty-six (31.7%) protocol patients died during their hospital stay compared to sixty-nine (47.6%) control patients (p=0.006). When all statistically and clinically relevant variables were controlled for, protocol patients had an adjusted odds ratio of dying in hospital of 0.429 (Cl 95% 0.213-0.864 p =0.018) when compared to the control group (Table 5). The relative risk reduction was found to be 33.3%.

## 4. Discussion

This study attempts to evaluate the utility and efficacy of using EGDT-sepsis based protocol on sepsis management at a tertiary care centre in Beirut. The primary outcome is in-hospital mortality; the secondary outcomes were 28-day mortality, ED, and hospital length of stay. After the introduction of the EGDT in the USA, clinical outcomes of septic patients substantially improved [7] and mortality from sepsis decreased [16, 17]. Novel interventions ranged from prehospital recognition and management of sepsis [18] to in-hospital optimization of care [6, 19, 20]. In Lebanon, data from national registries on mortality secondary to sepsis are still lacking. And the guidelines from the Surviving Sepsis Campaign are not adopted nationally, where the standard of care remains ill-defined [21]. To our knowledge, our study is the first to assess the impact of implementing EGDT sepsis based protocol in the emergency department on mortality outcomes in Lebanon. Our results show lower in-hospital mortality from sepsis after the introduction of the protocol, 31.7% compared to 47.6% when on “usual care,” before the protocol implementation. These findings are similar to the hospital mortality noted in the first EGDT paper (30.5% and 46.5% respectively) but higher than those reported in the more recent ProCESS, ARISE, and ProMISe trials [11]. Prehospital management of sepsis has a contributing impact on ultimate outcomes [22] and Lebanon lacks the infrastructure for prehospital sepsis management due to limited resources [23]. We assume that mortality is higher in our cohort compared to developed countries because prehospital care in Lebanon is not optimized [24].

### 4.1. Effectiveness of the Bundle

Adherence to the sepsis protocol was associated with a relative risk reduction (RRR) on in-hospital mortality of 33.3%. This is in line with the findings of the Rivers [7] and others [25] where the RRR ranged from 24.3% to 45% [11]. Our protocol-based care was associated with an adjusted odds ratio (AOR) for hospital mortality of 0.429 (Cl 95% 0.213-0.864 p =0.018).

Several studies questioned the effectiveness of the EGDT protocol versus standard care and showed that alternate strategies may have equal effect at reducing mortality without the increased costs [8–10]. Those studies highlight the need for “unbundling” the sepsis protocol to better define which intervention is most important while gauging specific clinical endpoints. A “single intervention” analysis in our paper is not possible as controlling for other interventions is not feasible. we noticed a more conservative approach to CVC placement in our study in both arms, the intervention and the control, 21.2% and 9.9%, respectively, compared to that reported in the EGDT and other trial were more than 50% of the control population had a CVC inserted [8]. This lower rate is potentially explained by the stipulated endpoints in our protocol: MAP (via non-invasive blood pressure readings) and urine output. While CVP measurement via CVC insertion was kept an optional clinical decision, our physicians were most of the times not driven by the indication of getting a CVP measurement to insert a CVC. This approach was supported by a meta-analysis that included 24 studies looking at the role of CVP measurement on fluid responsiveness and found a poor association [26]. Despite the difference in invasive monitoring rates in our study and other trials, mortality was reduced and at a comparable RRR. This finding might support the “unbundling” approach to invasive monitoring in resources-limited settings where cost and resources may become restrictive.

### 4.2. ED Length of Stay

Patient's length of stay in the ED and delayed transfers to the ICU have come under discussion in recent years [22, 26]. Some proposals have targeted ED times, such as the 4-hour rule [27], and other studies have shown an increased hospital stay and mortality with delayed patient transfer from the emergency department to the ICU [28, 29]. However, the length of stay in the ED in our study in both groups, the protocol and the control, was longer than that in the literature, 26.0 ± 29.0 hours and 19.4 ± 28.8 hours, respectively. This finding can be partially explained by the hospital's limited capacity of ICU beds [21]. In addition, the protocol arm had longer ED stay than the control arm, but it was not statistically significant. We explain this observed difference by the new introduction of the protocol in our ED, as the staff was in the training process to acquire the skills of the formulated protocol [30]. Moreover, the implementation of the new sepsis bundle was more time consuming, as it requires the use of more resources such as IV resuscitating fluids, vasopressors, monitoring, CVC placement and waiting for laboratory and radiology results. Delays in patient transfer from the ED to ICU in sepsis are well studied and correlate usually with worse outcomes. Despite the observed longer stay in the ED in our study, the mortality rate was still reduced at a comparable relative risk reduction to the EGDT trial.

### 4.3. Antibiotic Therapy

Our study showed that fewer patients were started on antibiotics in the ED in the protocol arm despite a lower mortality in this group. This finding relates to ongoing antimicrobial stewardship efforts [30]. It is crucial to promptly initiate antibiotic therapy in septic patients [31]; however, it is vital to apply standards of antimicrobial stewardship [32] as we are losing the battle against multidrug resistant organisms [33]. By following the protocol for sepsis management, we circumvented the initiation of unnecessary antibiotics. Though it did not reach significance, antibiotics were more used appropriately in the protocol arm than the control arm 91 (97.8%) vss. 75 (91.5%) (p=0.056). This reduction in inappropriate empiric antibiotic use might reflect positively on the cost, potential side effects, and complications. However, this study was not powered to test this hypothesis and we believe that future research in this direction would provide data to the gap in this knowledge.

## 5. Strengths and Limitations

This is the first study to be conducted in Lebanon assessing the effect of implementing an EGDT sepsis based protocol in ED on mortality outcome. The retrospective design of the study is a main limitation of the study as data collected from the electronic health records may sometimes be misinterpreted or missing. To minimize the information bias, frequent meetings were held between the investigators to standardize the data collection process. In addition, we were not able to use a matched control group. We included all patients who presented with sepsis between January 2013 and May 2014 to ensure a large sample size.

The definition of “usual care” is not standard and was per the treating physician, thus comparing the management across the two arms is limited. The control group in our study did receive timely antibiotics, vasopressors, and IV fluids. Data about the paramedical care was not available; therefore we were not able to adjust for it in our analyses. The two cohorts were not matched, and the control arm subjects were sicker at baseline, showing worse hemodynamics upon presentation to the ED. These points might be explained by the inaccuracy of the non-invasive measurements. They had lower diastolic blood pressure (DBP=54.7 ±16.0 mmHg vs. 59.2 ± 16.3 mmHg p=0.019) and mean arterial pressure (MAP= 73.7 ±17.3 mmHg vs. 69.5 ± 17.6 mmHg p=0.043) when compared to the intervention arm. These findings might not be pertinent clinically as both groups had similar lactate and arterial pH. Those markers are indicators of end-organ hypoperfusion [34]. Moreover, despite less fluid and vasopressor use in the control arm, this group had higher MAP after 6 hours of resuscitations (73.9 ± 14.9 mmHg vs. 69.8 ± 13.8 mmHg, respectively, p=0.022). However, patients of the control cohort had more severe kidney injury at baseline compared to intervention cohort, with higher creatinine (2.8 ± 2.2 mg/dl vs. 2.0 ± 1.4 mg/dl, respectively, p=<0.001) and BUN (55.9 ± 39.5 mg/dl vs. 41.9 ± 29.9 mg/dl, respectively, p=0.001). The internal validity of our study is limited by the missing data on the urine output, that was used as an endpoint in the sepsis protocol and the units of blood transfusions received in both groups. It is unclear which parts of the bundle contribute to the noted difference in mortality between the two groups. There is continued work in developed countries to decipher the most impactful parts of the EGDT protocol. In countries with limited resources, it is potentially more critical to determine those fundamental factors in order to decrease the effort and resources required to effectively treat sepsis. Finally, our study is not generalizable, as it is limited to one center in Lebanon and further multicenter studies are needed.

## 6. Conclusion

In a single tertiary center in Lebanon, the introduction of an EGDT-based sepsis protocol decreased in-hospital mortality of patients presenting with severe sepsis or septic shock. 28-day sepsis mortality is also reduced after the implementation of the EGDT protocol. Although the utility of EGDT bundles has been under scrutiny in recent years, their benefits when used in countries with limited resources, ICU capabilities and pre-hospital systems may be pronounced. In conclusion, this study highlights several potentially important points. The introduction of a structured approach to sepsis is feasible in a resource limited setting; the results achieved in terms of reduced mortality are comparable to those demonstrated in developed countries. Outcomes could be further improved, especially regarding the baseline mortality by improving the existing pre-hospital infrastructure to ensure expedited and appropriate treatment of septic patients.

## Figures and Tables

**Table 1:** Patient demographics.

	**Protocol N= 145**	**Control N= 145**	**p-value**

**Continuous mean ± SD**			

Age (years)	71.9 ± 14.1	72.9 ± 16.3	0.573

**Categorical no.(**%**)**			

Male	75 (51.7)	76 (52.4)	0.906

Diagnosis			0.280
Septic shock	92 (63.4)	83 (57.2)	
Severe sepsis	53 (36.6)	62 (42.8)	

HTN	102 (70.3)	101 (69.7)	0.898

DM	71(49.0)	68 (46.9)	0.724

CAD	64 (44.1)	59 (40.7)	0.552

Systolic CHF: EF<40%	32 (22.1)	29 (20.0)	0.666

COPD/Emphysema	13 (9.0)	19 (13.1)	0.261

CKD on HD	4 (2.8)	5 (3.4)	0.735

CVA	19 (13.1)	20 (13.8)	0.863

Site of Infection			<0.001^∗^
Lung	61 (42.1)	45 (31.0)	
Gastrointestinal	21 (14.5)	11 (7.6)	
Urine	52 (35.9)	49 (33.8)	
Skin	6 (4.1)	19 (13.1)	
Bile	0 (0.0)	5 (3.4)	
Liver	0 (0.0)	1 (0.7)	
Undetermined	5 (3.4)	15 (10.3)	

Microbiology Isolate			

CoNS^1^	1 (0.7)	2 (2.1)	0.562

Staphylococcus aureus	6 (4.1)	3 (2.1)	0.310

Escherichia coli	44 (37.9)	61 (42.1)	0.472

klebsiella pneumonia	9 (6.2)	11 (7.6)	0.643

pseudomonas aeroginosa	12 (8.3)	7 (4.8)	0.235

Acinetobacter baumani	8 (5.5)	5 (3.4)	0.395

Enterococcus spp.	2 (1.4)	9 (6.3)	0.030^∗^

Proteus mirabillis	4 (2.8)	7 (4.8)	0.356

Streptococcus spp.	9 (6.2)	6 (4.1)	0.426

Clostridium spp.	3 (2.1)	1 (0.7)	0.314

Others^2^	3 (2.1)	0 (0.0)	0.281

^1^Coagulase-negative staphylococci. ^*2*^*Others included: Bacteroides fragilis, Candida albicans, Citrobacter, Diphteroids *spp.,* Enterobacter cloacae, Haemophilus influenzae* (type B), *Haemophilus parainfluenzae, Legionella pneumophila, *Leuconostoc*, Morganella morgani, Peptococcus *spp.,* Providncia stuartii, Serratia marsescens, *and* Stenotrophomonas maltophilia*.

^∗^p=<0.05 considered significant.

**Table 2:** Preresuscitation parameters.

	**Protocol N= 145**	**Control N= 145**	**p-value**

**Continuous mean ± SD**			

SBP (mmHg)	104.7 ± 23.9	100.0 ± 26.0	0.114

DBP (mmHg)	59.2 ± 16.3	54.7 ± 16.0	0.019^∗^

MAP (mmHg)	73.7 ± 17.3	69.5 ± 17.6	0.043^∗^

HR (bpm)	106.2 ± 24.2	99.4 ± 25.3	0.021^∗^

O_2_ Saturation (%)	92.8 ± 8.5	93.4 ± 7.1	0.533

Temperature (°C)	37.7 ± 1.2	37.4 ± 1.4	0.071

RR (breath/min)	23.9 ± 7.2	22.9 ± 6.4	0.196

Glucose (mg/dl)	169.8 ± 102.9	172.3 ± 116.1	0.860

WBC (x10^9^cells/L)	15,238.6 ± 11,683.5	16,412.4 ± 15,648.1	0.470

Hemoglobin (g/dL)	11.0 ± 62.2	11.0 ± 2.3	0.935

Hematocrit(%)	33.1 ± 6.9	33.1 ± 7.9	0.994

Bicarbonate(mmol/L)	20.5 ± 6.0	19.9 ± 8.9	0.515

BUN (mg/dL)	41.9 ± 29.9	55.9 ± 39.5	0.001^∗^

Creatinine (mg/dL)	2.0 ± 1.4	2.8 ± 2.2	<0.001^∗^

Arterial pH	7.34 ± 0.1	7.34 ± 0.1	0.964

INR	1.8 ± 1.1	2.0 ± 1.3	0.397

Lactate^1^ (mmol/L)	3.9 ± 2.5	3.8 ± 4.1	0.787

Lactate > 4 mmol/L	18 (12.4)	48 (33.1)	0.190

^1^133 protocol patients had their lactate taken vs 67 controls.

^∗^p=<0.05 considered significant.

**Table 3:** Resuscitation parameters.

	**Protocol N= 145**	**Control N= 145**	**p-value**

***Vital signs after 6 hours***			

**Continuous mean ± SD**			

SBP (mmHg)	103.3 ± 19.9	104.7 ± 18.9	0.595

DBP (mmHg)	57.2 ± 13.0	59.1 ± 14.5	0.243

MAP (mmHg)	69.8 ± 13.8	73.9 ± 14.9	0.022^∗^

HR (bpm)	92.4 ± 21.0	91.9 ± 20.0	00.834

O2 Saturation (%)	96.0 ± 11.2	97.1 ± 6.8	0.394

Temperature (°C)	37.3 ± 0.9	37.4 ± 5.9	0.781

RR (breath/min)	21.8 ± 5.5	21.0 ± 4.3	0.166

***Resources variables***			

**Continuous mean ± SD**			

IV fluid requirements at first 6 hrs (L)	3.8 ± 1.7	2.7 ± 2.0	<0.001^∗^

IV fluid requirements at first 24 hrs (L)	6.1 ± 2.1	4.9 ± 2.8	<0.001^∗^

Time to initiation of antibiotics (hrs)	2.0 ± 3.6	2.8 ± 2.6	0.054

Time to vasopressor/inotrope use within the 1st 24hrs (hrs)	9.1 ± 22.6	7.7 ± 7.0	0.67

Duration of vasopressor/inotrope treatment within 1st 24hrs (hrs)	50.9 ± 649.6	40.0 ± 56.1	0.234

**Categorical No (**%**)**			

Vasopressor/inotrope use within 1st 24hrs	88 (60.6)	52 (35.8)	<0.001^∗^

CVC placement	29 (21.2)	14 (9.9)	0.010^∗^

Mechanical ventilation	31 (21.5)	23 (15.9)	0.217

Steroid use	50 (34.5)	54 (37.2)	0.624

Antibiotic use	143 (98.6)	145 (100.0)	0.155

Appropriate antibiotic use^1^	91 (97.8)%	75 (91.5)	0.056

Antibiotics initiation in the ED	136 (93.8)	144 (99.3)	0.010^∗^

Antibiotics initiation in the ICU	4 (2.8)	1 (0.7)	0.176

Antibiotics initiation in the GPU	3 (2.1)	0 (0)	0.082

^1^Appropriate use of antibiotics was defined as preliminary antibiotic given in the first 48hrs of treatment covering the bacteria grown later in bacteriology.

**Table 4:** Length of stay and mortality outcomes.

	**Protocol N= 145**	**Control N= 145**	**P-value**

**Length of Stay (mean ± SD)**			

ED (hours)	26.0 ± 29.0	19.4 ± 28.8	0.051

ICU (days)	5.8 ± 6.9	12.0 ± 38.9	0.285

GPU (days)	7.02 ± 6.2	7.1 ± 3.8	0.913

Hospital^1^ (days)	10.7 ± 8.7	15.3 ± 29.8	0.148

**Mortality no.(**%**)**			

In-hospital	46 (31.7)	69 (47.6)	0.006^∗^

72-hour	17 (11.7)	11 (7.6)	0.233

28-day^2^	30 (20.7)	47 (32.4)	0.014

^1^Hospital LOS days were calculated only for those that did not expire in hospital (as shorter LOS times may be associated with early deaths). ^2^28 patients (19.3%) and 14 (9.7%) of patients had unknown 28-day mortality in the protocol and control groups, respectively.

^∗^p=<0.05 considered significant.

**Table 5:** Multiple logistic regression for hospital mortality.

	**Protocol N= 145**	**Control N= 145**		

			**Adjusted OR** ^**2**^ ** (CI 95**%**)**	**P-value**

**Hospital mortality no.(**%**)**^**1**^	46 (31.7)	69 (47.6)	0.429 (0.213-0.864)	0.018^∗^

^1^Reference group is being the Control group.

^2^While controlling for the following: age, gender, diagnosis (severe sepsis or septic shock), systolic CHF: EF<40%, DM, CAD, HTN, cerebrovascular accidents, CKD, CKD on HD, COPD emphysema, CVC placed, ET tube placed, MAP upon presentation to the ED, BUN, creatinine, and appropriate use of antibiotics.

^∗^p=<0.05 considered significant.

## Data Availability

The protocol dataset applied in the intervention arm, used to support the findings of this study, is included within the supplementary material.

## Abbreviations

BP: Blood pressure
BUN: Blood urea nitrogen
CAD: Coronary artery disease
CHF: Congestive heart failure
COPD: Chronic obstructive pulmonary disease
CVC: Central venous catheter
CVP: Central venous pressure
DBP: Diastolic blood pressure
DM: Diabetes mellitus
ED: Emergency Department
EGDT: Early Goal Directed Therapy
EHR: Electronic health record
EM: Emergency medicine
ET: Endotracheal tube
GPU: General practice unit
HR: Heart rate
HTN: Hypertension
ICU: Intensive care unit
INR: International normalised ratio
LOS: length of stay
MAP: Mean arterial pressure
RR: Respiratory rate
RRR: Relative risk reduction
SBP: Systolic blood pressure
SSC: Surviving Sepsis Campaign.

## Supplementary Materials

Supplementary Materials Sepsis protocol order set that was used in the case arm.
