# Supplementary material for: The Impact of EGDT on Sepsis Mortality in a Single Tertiary Care Center in Lebanon
Source: Emerg Med Int. 2019 Jan 15;2019:8747282. doi: 10.1155/2019/8747282 (PMC6378781; doi:10.1155/2019/8747282)
Supplement: Supplementary Materials — Sepsis protocol order set that was used in the case arm. [file 8747282.f1.docx]

**Supplement 1: Sepsis Protocol Order Set**

|  | |  | | |
| --- | --- | --- | --- | --- |
| **Severe Sepsis – Order Set** | |  |  |  |
| **Last Name:** | | **Unit:** | | |
| **First and Middle Name: Patient Number:** |  | **Weight: Height:** | | |
|  |  | **Expanded Precautions:** 🗀 None 🗀 Airborne 🗀 Droplet  🗀 Contact 🗀 Contact Plus  **Other Precautions:** | | |
| **Date of Birth: Age:** | |  |  |  |
| **Gender: 🗀 Male 🗀 Female Admission Date:** | |  |  |  |
|  |  |  | | |
|  |  | **Allergy □ No □Yes (specify reaction):** | | |
| **Admitting Physician:** | |  |  |  |
|  | |  |  |  |
| **The following abbreviations may not be used to document patient care: U IU QD QOD .X mg X.0 mg MS MSO4 MgSO4 CC µg mcg**  **🙷 Check the Applicable Order** | | | | |
| **Unit:** 👎ED 👎ICU 👎General floor | | | **Nurse’s  Name and  Signature** | **Date &  Time  Noted** |
| 1. **Diagnosis:** | Refer to *Early Goal Directed Therapy Inclusion Criteria* Form. **Condition**: Critical | |  |  |
| 1. **Activity:** | 🙷Complete bed rest | |  |  |
| 1. **Vital Signs:** | 🙷Continuous cardiac monitor  🙷Monitor BP, HR, RR, SaO2, Temperature and record every 1 hour ~Apply supplemental O2 at ______liters per minute by ______ | |  |  |
| 1. **STAT Labs and Diagnostics:** | 🙷CBC 🙷Chem9 🙷PTT/INR 🙷Glucose 🙷Urinalysis 🙷CXR 🙷EKG  🙷Lactate STAT and in 3 hours ~ABG ELipase ELFTs ~D-Dimer ElFibrinogen ElTroponin, CK, CKMB STAT and in 8 hours | |  |  |
| 1. **Imaging:** | ElCT scan brain ElCT scan chest ElCT scan abdomen/pelvis El Echocardiogram ElOther: | |  |  |
|  |  | |  |  |
| 1. **Cultures:** | 🙷Blood cultures x 2 sets drawn STAT 🙷Urine  **Note:** At least one set from a peripheral stick indwelling vascular access  **Note**: If patient unable to provide urine within notify MD | 🙷Sputum ElOther |  |  |
|  |  | and at least one set from any  30 minutes of protocol initiation, |  |  |
| 1. **Fluids per IV:** | 🙷Insert two peripheral IV lines ElNSS 1000ml IV over 30 minutes ElNSS 2000ml IV over 30 minutes  ElOther: at ml per hour over | |  |  |
|  |  | |  |  |
| 1. **Intake/Output (I/O):** | 🙷Document I/O every hour ElInsert indwelling urinary catheter | |  |  |
| 1. **Nutrition:** | ElNPO ElInsert NG tube | |  |  |
| 1. **Antipyretics:** | ElPerfalgan 1 gram IV drip PRN every 6hours for rectal temperature greater than 38.5°C (Max dose 4 grams in 24 hours. Adjust dose for patients in renal or liver failure) | |  |  |

Revised February 2015 1/3

| **The following abbreviations may not be used to document patient care: U IU QD QOD .X mg X.0 mg MS MSO4 MgSO4 CC µ mc**  **.X mg X.0 mg MS MSO4 MgSO4 CC μg mcg** | | | | | |
| --- | --- | --- | --- | --- | --- |
| t D 0  **Allergy (specify reaction):** | | | | |  |
| **🙷 Check the Applicable Order** | | | **Name and  Signature** | **Noted** |  |
| 1. **Antibiotics:** (categories can be crossed) Creatinine ______ GFR | **Choose one of the below regimens** | |  |  |  |
|  | **Empiric Coverage** | ElPiperacillin/Tazobactam 4.5 grams IV drip every 6 hours  **OR**  ElCefepime 2 grams IV drip every 8 hours  **If extended-spectrum beta-lactamase (ESBL)** |  |  |  |
| **Goal** is to start antibiotics after blood cultures, within **first hour** of initiation of protocol, and **3 hours of ED**  **arrival**.  **Regimens are for patients with normal renal function and Cr Clearance greater than 50ml per minute** |  |  |  |  |  |
|  |  | **producing organisms are suspected:** |  |  |  |
|  |  | ElMeropenem 1 gram IV drip every 8 hours |  |  |  |
|  | **Add the following if: Source is pulmonary and coverage**  **needed for atypical pathogens** | ElLevofloxacin 750 mg IV drip once daily **OR**  ElMoxifloxacin 400 mg IV drip once daily |  |  |  |
|  | **Add the following if: Double coverage for multi-drug resistant (MDR) pathogens or Pseudomonas^1^ is suspected1** | ElAmikacin 1000mg IV drip once daily |  |  |  |
|  | **Add the following if: Septic shock,**  **indwelling catheter, or history of methicillin- resistant**  **Staphylococcus**  **aureus (MRSA)** | ElVancomycin 1gram IV drip every 12 hours |  |  |  |
| 1. **Consult:** | 🙷Infectious Disease consult after first dose of antibiotics | |  |  |  |
|  | 🙷 Critical Care Consult El Other: | |  |  |  |
| **Identifying**  **patients at risk for MDR** | **Patients at risk for multi-drug resistant (MDR) pathogens or Pseudomonas:** | |  |  |  |
|  | D Hospitalized in an acute care hospital for 2 or more days in the preceding 90 days  D Resided in a nursing home or long-term care facility in the  preceding 30 days  D Received intravenous antibiotic therapy or intravenous chemotherapy in the preceding 30 days  D Attended a hospital or hemodialysis unit in the preceding 30 days  D Received wound care in the preceding 30 days  D Have a history of multi-drug resistant pathogen  D There is a high frequency of antibiotic resistance in the community, long-term care facility or hospital unit | |  |  |  |

Revised February 2015 2/3

| **The following abbreviations may not be used to document patient care: U IU QD QOD .X mg X.0 mg MS MSO4 MgSO4 CC µg mcg** | | | | |
| --- | --- | --- | --- | --- |
| **Allergy (specify reaction):** | | | | |
| **RI Check the Applicable Order** | | | | |
| 1. **Central Line:** | ElSet up for insertion of central line | | | |
| 1. **Early Goal Directed Therapy**   **(EGDT)**  **Goal** is to achieve the following  **within first 6 hours** of identification:   - Urine output greater than or equal to 0.5 ml per kg per hr - Mean Arteri al Pressure(MAP) greater than or equal to 65 mmHg | **Fluids** | ElMonitor Central Venous Pressure (CVP) continuously and record every hour **(not applicable to patients on general floors)** |  |  |
|  |  | ElNSS 0.9% 1000ml/hour  El Other: Rate ml per hr |  |  |
|  |  | Notify MD if 6 liters of crystalloids have been administered |  |  |
|  | **Vasopressors** | ElIf Mean Arterial Pressure (MAP) is less than 65mmHg despite adequate fluid therapy, initiate vasopressors |  |  |
|  |  | ElTitrate as ordered to a MAP greater than or equal to 65 mmHg |  |  |
|  |  | ElNorepinephrine (Levophed) IV drip: 0-20 micrograms per minute IV continuous infusion  Start at ______ microgram(s) per minute  Titrate to MAP greater than or equal to 65mmHg per vasopressors protocol Hold if Systolic BP greater than ________  **☜☪If dose reaches 10 micrograms/minute MAP is less 65 mmHg notify MD on**  **call**  **.**  **☜☪ Please nobfy MD on call if dose exceeds 20microgram per minute** |  |  |
|  |  | ElOther: IV drip dose: |  |  |
|  |  | Start at |  |  |
|  |  | Titrate to MAP greater than or equal 65mmHg per protocol Hold if Systolic BP greater than |  |  |
|  |  | Instructions: Please notify MD on-call if dose exceeds: |  |  |
|  |  |  |  |  |
| 1. **Blood**   **Products**  (Target Hemoglobin greater than or  equal to 7gr per deciliter) | ElType and cross ______ units packed RBC | |  |  |
|  |  | ElTransfuse  unit packed RBC over hours each  _______ |  |  |
|  |  |  |  |  |
| 1. **Peptic Ulcer Disease Prophylaxis:** |  | ElPantozol  ElOther:(pantoprazole) 40 mg IV drip every ______ hours |  |  |
|  |  |  |  |  |
| 1. **Venous Thromboemb olism (VTE) Prophylaxis:** | El | ElLow-Molecular Weight Heparin  Name Dose: (El mg; El units; El ml) |  |  |
|  |  | Subcutaneous every |  |  |
|  |  | **OR** |  |  |
|  |  | ElHeparin  ElOther:  5000 units subcutaneously every 12 hours |  |  |
|  |  | ElSequential Compression Device |  |  |
| 1. **Glucose Control:** | El Blood glucose finger sticks every ______ hours  ElInitiate Adult Hypoglycemia Management Protocol (if applicable) El Initiate Adult Hyperglycemia Protocol (if applicable) | |  |  |
| 1. **Sedation:** | ElInitiate Pain/ Sedation Protocol (if applicable) | |  |  |

Revised February 2015 3/3
